# Correction to: Monitoring activities of receptor tyrosine kinases using a universal adapter in genetically encoded split TEV assays

**DOI:** 10.1007/s00018-019-03244-9

**Published:** 2019-08-03

**Authors:** Jan P. Wintgens, Sven P. Wichert, Luksa Popovic, Moritz J. Rossner, Michael C. Wehr

**Affiliations:** 1 Department of Psychiatry and Psychotherapy, University Hospital, LMU Munich, Nussbaumstr. 7, 80336 Munich, Germany; 2 Systasy Bioscience GmbH, Adams-Lehmann-Str. 56, 80797 Munich, Germany

## Correction to: Cellular and Molecular Life Sciences (2019) 76:1185–1199 10.1007/s00018-018-03003-2

The article Monitoring activities of receptor tyrosine kinases using a universal adapter in genetically encoded split TEV assays, written by Jan P. Wintgens, Sven P. Wichert, Luksa Popovic, Moritz J. Rossner and Michael C. Wehr, was originally published electronically on the publisher’s internet portal (currently SpringerLink) on 8 January 2019 without open access.

With the author(s)’ decision to opt for Open Choice the copyright of the article changed on 1 August 2019 to © The Author(s) 2019 and the article is forthwith distributed under the terms of the Creative Commons Attribution 4.0 International License (http://creativecommons.org/licenses/by/4.0/), which permits use, duplication, adaptation, distribution and reproduction in any medium or format, as long as you give appropriate credit to the original author(s) and the source, provide a link to the Creative Commons license and indicate if changes were made.

